# Exercise intensity effects on total sweat electrolyte losses and regional vs. whole-body sweat [Na^+^], [Cl^−^], and [K^+^]

**DOI:** 10.1007/s00421-018-4048-z

**Published:** 2018-12-06

**Authors:** Lindsay B. Baker, Peter John D. De Chavez, Corey T. Ungaro, Bridget C. Sopeña, Ryan P. Nuccio, Adam J. Reimel, Kelly A. Barnes

**Affiliations:** 1 Gatorade Sports Science Institute, 617 W. Main St., 60010 Barrington, IL USA; 2 PepsiCo R&D, Barrington, IL USA

**Keywords:** Absorbent patch, Body map, Chloride, Potassium, Sodium

## Abstract

**Purpose:**

To quantify total sweat electrolyte losses at two relative exercise intensities and determine the effect of workload on the relation between regional (REG) and whole body (WB) sweat electrolyte concentrations.

**Methods:**

Eleven recreational athletes (7 men, 4 women; 71.5 ± 8.4 kg) completed two randomized trials cycling (30 °C, 44% rh) for 90 min at 45% (LOW) and 65% (MOD) of *V*O_2max_ in a plastic isolation chamber to determine WB sweat [Na^+^] and [Cl^−^] using the washdown technique. REG sweat [Na^+^] and [Cl^−^] were measured at 11 REG sites using absorbent patches. Total sweat electrolyte losses were the product of WB sweat loss (WBSL) and WB sweat electrolyte concentrations.

**Results:**

WBSL (0.86 ± 0.15 vs. 1.27 ± 0.24 L), WB sweat [Na^+^] (32.6 ± 14.3 vs. 52.7 ± 14.6 mmol/L), WB sweat [Cl^−^] (29.8 ± 13.6 vs. 52.5 ± 15.6 mmol/L), total sweat Na^+^ loss (659 ± 340 vs. 1565 ± 590 mg), and total sweat Cl^−^ loss (931 ± 494 vs. 2378 ± 853 mg) increased significantly (*p* < 0.05) from LOW to MOD. REG sweat [Na^+^] and [Cl^−^] increased from LOW to MOD at all sites except thigh and calf. Intensity had a significant effect on the regression model predicting WB from REG at the ventral wrist, lower back, thigh, and calf for sweat [Na^+^] and [Cl^−^].

**Conclusion:**

Total sweat Na^+^ and Cl^−^ losses increased by ~ 150% with increased exercise intensity. Regression equations can be used to predict WB sweat [Na^+^] and [Cl^−^] from some REG sites (e.g., dorsal forearm) irrespective of intensity (between 45 and 65% *V*O_2max_), but other sites (especially ventral wrist, lower back, thigh, and calf) require separate prediction equations accounting for workload.

## Introduction

Thermoregulatory sweat is comprised of several electrolytes, including sodium (Na^+^), chloride (Cl^−^), and potassium (K^+^). It is well established that sweat electrolyte, particularly Na^+^ and Cl^−^, losses during exercise vary considerably within and between athletes (Baker et al. 2016; Sawka et al. 2007). One reason for this variability is exercise intensity differences across training sessions/competition. Sudomotor output, as indicated by both whole-body sweating rate (WBSR) and regional sweating rate (RSR), increases proportionally with the rate of metabolic heat production (Gagnon et al. 2013; Smith and Havenith 2011, 2012). In addition, previous studies have shown that regional (REG) sweat [Na^+^] increases with higher metabolic rates: as sweat flow rate increases Na^+^ secretion rate increases proportionally more than the Na^+^ reabsorption rate (Buono et al. 2008). However, few have studied the effect of exercise intensity on WB sweat [Na^+^], [Cl^−^], and [K^+^] and total WB sweat electrolyte losses. In one study, Dill et al. (1966) measured WB sweat [Cl^−^] using the washdown technique in 12 men walking/running at various metabolic rates in a desert (34–47 °C). The authors (Dill et al. 1966) concluded that sweat [Cl^−^] tended to increase with sweating rate, but the extent of the increase in sweat [Cl^−^] and its relation to metabolic rate were not reported. Lemon et al. (1986) made WB sweat measurements in six men during treadmill exercise and found that sweat urea nitrogen loss increased with changes in intensity from 42 to 67% maximal oxygen uptake (*V*O_2max_); however, electrolytes were not measured in the WB sweat. Thus, more work is needed to determine WB sweat [Na^+^], [Cl^−^], and [K^+^] at various exercise intensities. Having a better understanding of how WB sweat electrolyte concentrations as well as WBSR are modified by workload can help to inform athletes’ fluid/electrolyte replacement strategies for different intensities of training/competition.

Sports practitioners and scientists commonly estimate sweat electrolyte concentrations via user-friendly REG sweat sampling techniques (e.g., absorbent patches) to help determine personalized fluid/electrolyte replacement strategies (Maughan and Shirreffs 2008, 2010). Several studies have described the relation between REG and WB sweat electrolyte concentrations (Baker et al. 2009; Patterson et al. 2000; Shirreffs and Maughan 1997). For instance, our group (Baker et al. 2018) recently measured the relation between WB and REG sweat [Na^+^], [Cl^−^], and [K^+^] at nine anatomical sites, and found that regression equations can be used to predict WB sweat electrolyte concentrations from REG with an acceptable level of accuracy (especially using the forearm or thigh). Furthermore, there were minimal effects of sex or day-to-day variability on the prediction models (Baker et al. 2018). However, the previous studies have compared REG vs. WB electrolyte concentrations at only one level of exercise intensity (Baker et al. 2009, 2018; Patterson et al. 2000; Shirreffs and Maughan 1997). Thus, it is unclear whether the regression equations relating REG-to-WB sweat [Na^+^] are consistent across multiple workloads.

Previous studies have investigated the effect of exercise intensity on the relation between REG sweating rate (RSR) and WBSR. Smith and Havenith have published comprehensive body maps of RSR in both men (Smith and Havenith 2011) and women (Smith and Havenith 2012), and reported little change in the distribution of sweating with a change in exercise intensity. Specifically, no significant differences in the ratio of RSR-to-area-weighted total sweat rate from all regions were observed between 55 and 75% *V*O_2max_ in men (Smith and Havenith 2011). Furthermore, of the 39 regions measured in the study with women, the only significant changes were an increase in sweat distribution toward the breasts and a decrease toward the feet and shoulders when increasing running workload from 60 to 75% *V*O_2max_ (Smith and Havenith 2012). No differences in the ratio of RSR-to-area-weighted total sweat rate from all regions were reported for women at the sites typically used for REG sweat electrolyte testing in the field (e.g., forearms, upper arms, upper/lower back, or thighs) (Smith and Havenith 2012). Since the regional distribution of sweat rate does not seem to be effected by changes in exercise intensity and sweat Na^+^ is determined primarily by sweating rate (within subjects), then one may hypothesize that the relation between REG and WB sweat electrolyte concentrations would also be generally unaffected by workload. However, empirical data are needed.

The purpose of this study was to measure sweat [Na^+^], [Cl^−^], and [K^+^] at both REG and WB levels during low (LOW) and moderate (MOD) relative exercise intensities to determine the effect of increasing workload on: (1) WB sweat electrolyte concentrations, (2) total sweat electrolyte losses, and (3) the relation between REG and WB sweat electrolyte concentrations. It was hypothesized that the REG and WB concentrations of sweat electrolytes, particularly Na^+^ and Cl^−^, would increase significantly, but the relation between REG and WB would not change significantly with an increase in exercise intensity.

## Methods

### Subjects

Eleven Caucasian healthy, recreational endurance athletes (7 men and 4 women) volunteered to participate in this study. Their age, body mass, height, maximal heart rate (HR_max_), *V*O_2max_, and fasting blood glucose were 34 ± 5 years, 71.6 ± 8.4 kg, 173 ± 6 cm, 184 ± 8 bpm, 50.6 ± 7.6 ml/kg/min, and 98 ± 8 mg/dl, respectively. This study was approved by the Sterling Institutional Review Board (Atlanta, GA; sterlingirb.com; an independent review board not affiliated with GSSI) and has, therefore, been performed in accordance with the ethical standards laid down in the 1964 Declaration of Helsinki. Participants were informed of the experimental procedures and associated risks before providing written informed consent. A portion of these data (MOD trials) were included in a previous publication describing the effect of sex and within-subject variability (bilateral and day-to-day) on the relation between REG and WB sweat responses (Baker et al. 2018).

### Preliminary screening measurements

After providing written informed consent, subjects’ nude-body mass, height, resting heart rate, resting blood pressure, and 8-h fasted blood glucose concentration were measured. During this preliminary screening visit, subjects also completed a graded exercise test to assess cardiovascular fitness (12-lead electrocardiogram (ECG), Schiller AT-10 Plus, Schiller America; Doral, FL), HR_max_, and *V*O_2max_ (MOXUS, AEI Technologies; Pittsburgh, PA) on a treadmill (Cosmed T200S, Cosmed USA; Chicago, IL). Main criteria for exclusion were: an abnormal resting or exercise ECG during the graded exercise test; smoking; asthma; pregnancy; or medical conditions or the taking of medications that may influence thermoregulatory or cardiovascular function.

### Experimental procedures for intensity comparison

In this study, each subject served as their own control and completed two experimental trials, which differed only in the intensity of exercise (LOW: 45% *V*O_2max_; and MOD: 65% of *V*O_2max_), in randomized counterbalanced order. The two trials were separated by at least 3 days, but no more than 15 days. The female subjects completed both trials in the luteal phase of their menstrual cycle. Previous studies have shown that sex has minimal effects on the relation between REG and WB sweat electrolyte concentrations (Baker et al. 2018). Thus, male and female subjects were included in the present study, so that results would be relevant to both sexes. All trials were completed in the late fall through winter months (November–March) in northeast Illinois. Therefore, subjects were not heat-acclimatized and this status remained consistent throughout their participation in the study.

For each trial subjects reported to the laboratory at 08:00 h or 13:00 h. The trial start times were consistent within subjects. Subjects were asked to abstain from caffeine, alcohol, and vigorous exercise in the 24 h preceding each trial. Two hours before the trials, subjects drank 500 ml of water, but fasted otherwise. In addition, subjects were asked to consume a consistent diet in the 48 h preceding each trial and record all food and fluid intake in that time frame. Diets were analyzed using Nutribase™ Software (Pro Edition v.11.64; CyberSoft, Inc.; Phoenix, AZ). Subjects were also asked to maintain a consistent training regimen throughout the duration of the study. A urine sample was collected for the assessment of baseline urine specific gravity (USG; Atago Pen Refractometer, 3741-E03, Tokyo Japan).

During the experimental trials, subjects cycled on a friction-braked ergometer (Monark model 828 E) in a plastic isolation chamber for 90 min in a warm environment. There were no differences in air temperature (LOW: 30.1 ± 0.3 °C; MOD: 30.1 ± 0.2 °C) or relative humidity (LOW: 43 ± 1%; MOD: 44 ± 1%) between LOW and MOD trials. Heart rate was monitored using telemetry (Polar Electro RS400; Lake Success, New York). For the purpose of this study, relative workloads were standardized across subjects to elicit LOW and MOD intensity exercise representative of a light training day (Bangsbo et al. 2006; DeMartini et al. 2011) versus moderate-intensity training/competition (Coutts et al. 2003; Krustrup et al. 2005; Narazaki et al. 2009; Padilla et al. 2000), respectively. This applied approach was chosen over standardizing absolute workload to increase the ecological validity and practical applicability of results. For example, it is expected that absolute workloads, and thus, SR will vary across individuals, especially between men and women, during real-world training/competition (Baker et al. 2016; Broad et al. 1996).

Ratings of perceived exertion [RPE, (Borg 1982)], ergometer resistance, and cadence were recorded every 10 min. Cycling work rate was used to calculate power output (watts) and energy expenditure (kcal) (ACSM 2014). Subjects drank a commercially available 6% carbohydrate–electrolyte (36 mmol/l Na^+^, 10 mmol/l K^+^) solution ad libitum during exercise. Fluid intake was measured as the difference in drink bottle mass from pre- to post-exercise to the nearest 0.01 g (Mettler Toledo, PG6002-S). Immediately before and after exercise, nude-body mass was recorded to the nearest 0.01 kg (Mettler Toledo KCC150 platform and IND690 reader; Columbus, OH). Subjects were asked to towel dry before each measurement.

### WBW sweat collection

The WBW method published and validated previously was used to determine WB sweat [Na^+^], [Cl^−^], and [K^+^] (Armstrong and Casa 2009; Dill et al. 1966; Lemon et al. 1986; Patterson et al. 2000). Recovery of sweat [Na^+^], [Cl^−^], and [K^+^] using the WBW method is 101%, 107%, and 96%, respectively (Baker et al. 2018). In addition, the reliability (coefficient of variation, CV) of the WBW method is 4%, 8%, and 18% for [Na^+^], [Cl^−^], and [K^+^], respectively (Baker et al. 2018).

Before entering the plastic isolation chamber, subjects were rinsed with 5.0 L of deionized water and then dried with electrolyte-free paper towels (Kimberly Clark Wypall L-40; Irving, TX). Next, subjects donned shorts/sport bra and heart rate monitor, which had been previously rinsed with deionized water to remove any electrolytes and air dried. The plastic isolation chamber consisted of a bale bag (Farm Bag Film Division, LLC; Glenford, OH) inside a metal frame (68 in × 48 in × 60 in). The cycle ergometer was washed with deionized water and air dried prior to placing it in the isolation chamber for each trial. During exercise, care was taken to avoid sweat dripping off the skin surface. Windows were cut out of the front and sides of the bag (18 in × 18 in) to promote evaporative cooling via external fans (frontal and side velocity of 2.8 m/s and 0.8 m/s, respectively). Excess sweat was wiped from the subjects face and torso with an electrolyte-free paper towel. At the end of exercise, subjects stepped off the ergometer and removed their clothing while still in the isolation chamber. The shorts/sport bra and paper towels used to wipe the subject’s sweat were hung to air dry. Then, the subject (nude) was rinsed thoroughly with 5.0 L deionized water to ensure removal of all sweat Na^+^, Cl^−^, and K^+^ from the skin and hair. After rinsing, the subject dried off with electrolyte-free paper towels and stepped out of the isolation chamber. The heart rate monitor and subject’s shorts/sport bra, as well as all paper towels, gauze, elastic netting, and gloves that touched the subject during the experiment were put in the bottom of the bale bag. After thoroughly mixing the contents collected at the bottom of the bale bag, a post-rinse sample was collected for electrolyte analysis via ion chromatography.

### Regional sweat collection

The subject’s skin was shaved and cleaned with alcohol at the patch locations before the pre-exercise whole-body rinse. Absorbent patches (3M Tegaderm™ + Pad; pad size 10 cm^2^ with an absorbent capacity of ~ 1.3 g) were used to collect sweat from the following anatomical sites: forehead, dorsal mid-forearm, ventral mid-forearm, dorsal wrist, ventral wrist, tricep, upper chest, scapula, lower back, ventral mid-thigh, and calf. These sites were chosen to represent a variety of upper and lower body areas that are relatively accessible from a practical perspective (for application of results to a real-world field setting). In addition, sites such as the wrists were included, because many of the prototype wearable devices aiming to measure sweat electrolytes or other biomarkers are wrist-worn devices (Gao et al. 2016; McCaul et al. 2018; Parrilla et al. 2016), yet little empirical sweat data are available from the wrists.

**Fig. 1:**
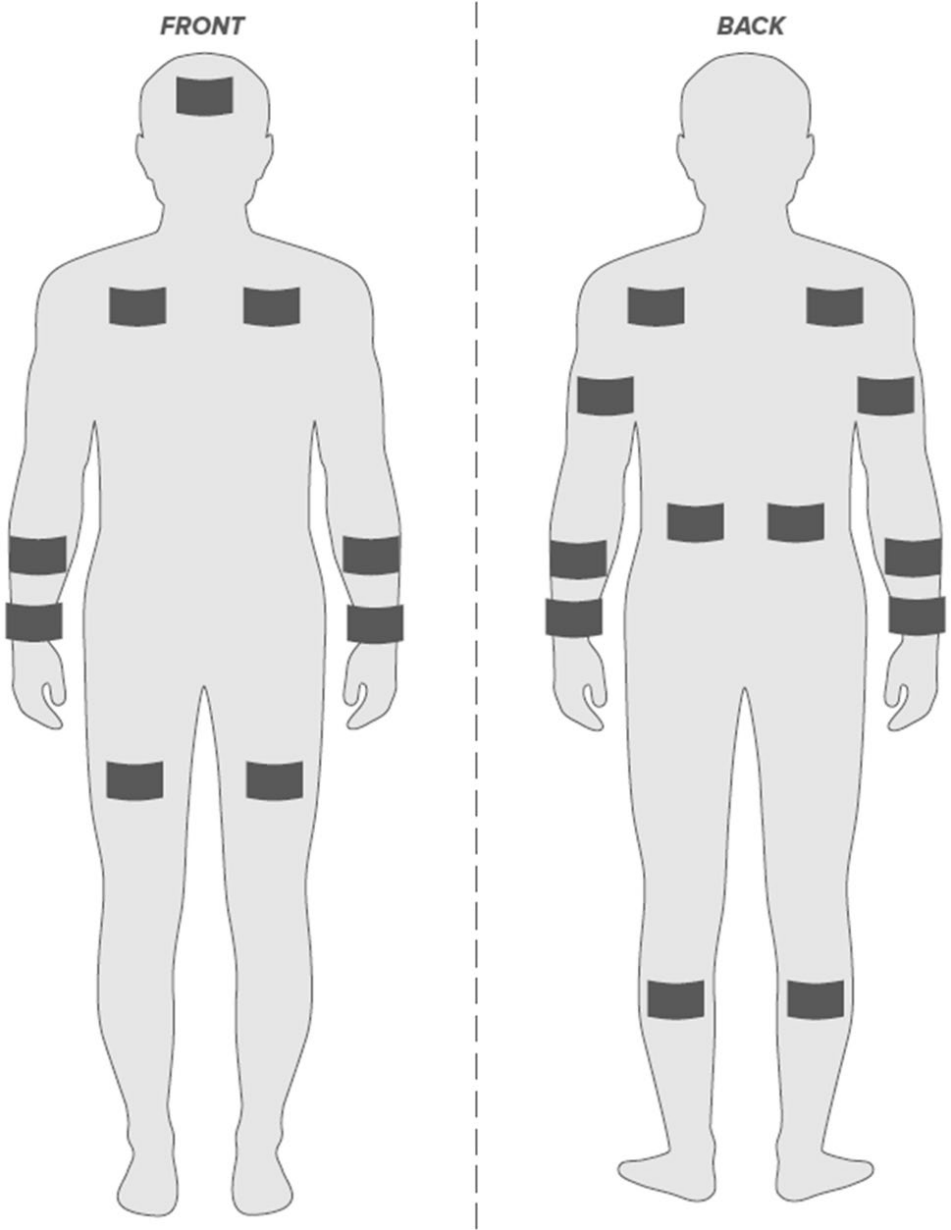
Body map showing the locations of regional patch placement

For all sites except the forehead, patches were placed on both the right and left sides for a total of 21 collection sites (see Fig. 1 for patch placement). Immediately prior to patch application, the skin was wiped dry with electrolyte-free gauze (4 in × 4 in; Thermo Fisher Scientific; Waltham, MA). Absorbent patch application began 15 min after the onset of exercise and was completed within 15 min. Patches were applied to the 21 anatomical sites in the same order (starting with the thigh and ending with the forehead) for each trial. Right and left patches on a given site and dorsal and ventral forearm patches were applied/removed consecutively. Patches were removed upon moderate sweat absorption (~ 0.5 g), but prior to saturation as determined by visual inspection. Upon removal, the absorbent pad was immediately separated from the Tegaderm™ using clean forceps and placed in an air-tight plastic tube (Sarstedt Salivette; Germany). The pad was centrifuged at 3000 rpm and 4 °C for 10 min to extract the sweat sample for subsequent analysis.

Target sweat volume for all patches was ~ 0.5 g (to collect ample volume for duplicate analysis of all electrolytes). Actual sweat absorption across all sites was 0.489 ± 0.113 g for LOW and 0.533 ± 0.144 g for MOD. Based on previous research, the within-subject within-site CV for sweat absorption between repeat trials was 15.7% (Baker et al. 2018). Because of interindividual variability in sweating rates, we could not standardize both pad absorption level and patch adherence time on skin. Therefore, we controlled for volume collected to avoid the confounding effects of absorption level (e.g., hidromeiosis) (Baker 2017) and ensure sufficient volume of sweat for electrolyte analysis. As expected, patch adherence time varied across sites. For example, during LOW, the forehead site (highest RSR) took 26.5 ± 13.7 min to absorb the target sweat volume, whereas the calf site (lowest RSR) took 59.8 ± 9.2 min. During MOD, the forehead site (highest RSR) took 14.3 ± 6.9 min to absorb the target sweat volume, whereas the calf site (lowest RSR) took 47.8 ± 14.0 min.

### Sweat analysis

On the same day as collection REG and WBW sweat samples were analyzed in duplicate for [Na^+^], [Cl^−^], and [K^+^] via ion chromatography (Dionex ICS-3000). Corrections to REG sweat [Na^+^] and [Cl^−^] were made according to a previous publication (Baker et al. 2018). Specifically, the background mmol/L of electrolytes in the absorbent patches was subtracted from the mmol/L value obtained from ion chromatography analysis. The following regression equations were used: (1) background [Na^+^] = − 4.377 ln (REG sweat mass in grams) + 4.300 (*r*^2^ = 0.98); (2) background [Cl^−^] = − 1.602 ln (REG sweat mass in grams) + 3.586 (*r*^2^ = 0.86). No corrections for [K^+^] were needed (Baker et al. 2018). The CV for measuring sweat electrolyte concentrations using the REG absorbent patch method is ~ 3–5% (Baker et al. 2018).

### Calculations

#### Body surface area

Body surface area was calculated from nude-body mass and height using the Dubois and Dubois equation (1916).

#### Whole-body sweat loss and sweating rate

WB sweat loss (WBSL) was calculated from the change in pre- to post-exercise nude-body mass, corrected for fluid intake, respiratory water loss, and weight loss due to substrate oxidation. Respiratory water loss and weight loss due to substrate oxidation were estimated using equations from ACSM (2014) and Mitchell et al. (1972). Subjects did not use the bathroom between the pre- and post-exercise nude body mass measurements. WBSR (in mg/cm^2^/min) was calculated by dividing WBSL by the subject’s body surface area and the duration of exercise (90 min).

#### Whole-body sweat electrolyte concentrations and total sweat electrolyte loss

WBW sweat [Na^+^], [Cl^−^], and [K^+^] were determined from dilution calculations based on the measured electrolyte concentrations in the post-rinse solution, the known volume of deionized water added to the bale bag, and WBSL. Total sweat Na^+^, Cl^−^, and K^+^ loss were determined from the product of WBSL and WB sweat [Na^+^], [Cl^−^], and [K^+^].

#### Regional sweating rate

RSR was measured gravimetrically based on the mass of sweat absorbed into the pad (using Mettler Toledo DeltaRange Precision Scales XS204 and AG204; Plano, TX), the patch surface area, and the duration that the patch was on the skin. Mean RSR was calculated from an aggregate of all 21 sites, which was weighted for body surface area using the following weighting coefficients (ISO 2004): forehead 0.07, chest 0.175, back 0.175, upper arms (triceps) 0.095, lower arms 0.095, and legs 0.39. Where the chest was the mean of two patches (right and left chest), the back was the mean of four patches (right and left scapula and lower back), the lower arms was the mean of eight patches (right and left dorsal wrist, ventral wrist, dorsal forearm, and ventral forearm), and the legs were the mean of four patches (right and left ventral thigh and calf).

#### Regional sweat electrolyte concentrations

Mean REG sweat [Na^+^], [Cl^−^], and [K^+^] were calculated from an aggregate of all 21 sites, which was weighted for RSR and body surface area using the same weighting coefficients listed for RSR above (ISO 2004).

#### Regional to whole-body ratios

Ratios were calculated in which REG values were expressed relative to corresponding WB values for SR and sweat electrolyte concentrations. Specifically, the REG value at a given anatomical site was divided by the WB value, such that a ratio < 1 indicates that REG was lower than WB, while a ratio > 1 indicates that REG was higher than WB.

### Statistical analyses

Analyses were carried out using Statistical Analysis Software version 9.4 (SAS Institute, Cary NC) and Minitab 17 Statistical Software (Minitab Inc., State College PA). The significance level for all statistical tests was set at *α* = 0.05. Kolmogorov–Smirnov tests were conducted to assess normality of the data. In instances of deviation from normality, data were natural-log transformed prior to running statistical tests. A power of 0.79–0.99 and 0.69–1.00 was achieved for the regression models predicting WB sweat [Na^+^] from REG for sweat [Na^+^] and [Cl^−^], respectively. Data are shown as mean ± standard deviation, unless otherwise indicated.

Based on paired t test analyses, there were minimal significant bilateral differences for REG sweat [Na^+^], [Cl^−^], and [K^+^] or RSR. Specifically, the only significant (*p* < 0.05) differences between right and left sides were for sweat [Na^+^] (*p* = 0.02, effect size, ES = 0.11) and [K^+^] (*p* = 0.04, ES = 0.31) on the dorsal forearm at LOW, sweat [Cl^−^] on the ventral wrist at MOD (*p* = 0.01, ES = 0.29), and RSR on the thigh at LOW (*p* = 0.04, ES = 0.42). Thus, the mean of right and left values was calculated for each subject, and used for all subsequent analyses and corresponding tables and figures. In instances of missing data from the right side or left side (due to inadequate sample volume or premature detachment of patch from skin; *n* = 11 out of 462 total samples), data from the one available patch were used in the analyses. For one male subject, both the right and left patches on the wrists became detached prematurely, and thus, *n* = 10 (instead of *n* = 11) for all analysis of dorsal and ventral RSR and REG sweat electrolyte concentrations.

Paired t tests were conducted to assess differences between LOW and MOD in descriptive measures, fluid balance, WBSR, WBSL, WB sweat electrolyte concentrations, total sweat electrolyte losses, 11-site aggregate RSR, and 11-site aggregate REG sweat electrolyte concentrations. A repeated-measures two-way analysis of variance (site by intensity) followed by Tukey’s HSD analysis (where main effects were found) was conducted to determine the effect of exercise intensity on: (1) RSR, (2) REG sweat electrolyte concentrations, (3) the ratio of RSR to WBSR, and (4) the ratio of REG-to-WB sweat electrolyte concentrations. ES were also calculated using Cohen’s* d* (Cohen 1988). An ES was defined as small when < 0.2, moderate when 0.2–0.8, and large when > 0.8.

To determine the relation between RSR and WBSR as well as REG and WB sweat electrolyte concentrations, simple linear regressions and Pearson product-moment correlations were conducted. The prediction strength of RSR-to-WBSR and REG-to-WB sweat electrolyte concentrations was assessed via the coefficients of determination (*r*^2^) of the linear regression models (Thomas and Nelson 2001). To assess the agreement between REG and WB measures, the slope (proportional error) and y-intercept (constant error) of the regression lines of the scatterplots of REG vs. WB were compared against the line of identity (slope of 1 and intercept of 0) (Westgard and Hunt 1973). In addition, intensity and intensity-by-REG interaction terms were included in the regression models of REG on WB to determine if exercise intensity had a significant effect on the prediction of WB from REG. Where applicable, non-significant intensity and interaction terms were dropped from the model and the model was reduced to include only REG and subject as a blocking factor (i.e., both LOW and MOD data included in one model).

## Results

### Baseline and general descriptive data

Baseline pre-exercise body mass (71.53 ± 8.40 kg vs. 71.58 ± 8.43 kg; *p* = 0.72, ES = 0.01) and USG (1.009 ± 0.006 vs. 1.009 ± 0.006; *p* = 0.89, ES = 0.03) did not differ between LOW and MOD trials. Table 1 shows sweat loss and other fluid balance variables during the LOW and MOD trials. Overall fluid balance, expressed as the percentage change in body mass from baseline, was significantly (*p* = 0.02, ES = 0.62) different between LOW and MOD trials. However, a 0.3% difference in body mass change is unlikely to impact study results (i.e., sweating rate and sweat electrolyte concentrations), and importantly, during both trials, all subjects maintained body mass well within 2% of that of baseline. Power output (109 ± 20 watts vs. 169 ± 27 watts; *p* < 0.0001, ES = 1.56), energy expenditure (722 ± 105 kcal vs. 999 ± 138 kcal; *p* < 0.0001, ES = 1.49), heart rate (68 ± 2% HR_max_ vs. 86 ± 2% HR_max_; *p* < 0.0001, ES = 1.90), intensity (48 ± 6% *V*O_2max_ vs. 66 ± 7% *V*O_2max_; *p* < 0.0001, ES = 1.64), and RPE (11 ± 1 vs. 14 ± 1.; *p* < 0.0001, ES = 1.71) were significantly lower during the LOW than the MOD trials. WBSR and WBSL were also significantly lower during LOW than MOD.

**Table 1:** Fluid balance

	Whole-body sweating rate (kg/h)	Whole-body sweating rate (mg/cm^2^/min)	Total whole-body sweat loss (kg)	Respiratory water loss (kg)	Metabolic mass loss (kg)	Ad libitum fluid intake (kg)	Fluid balance (% change in body mass)
LOW (*n* = 11)	0.57 ± 0.10*	0.516 ± 0.077*	0.86 ± 0.15*	0.09 ± 0.01*	0.08 ± 0.01*	0.518 ± 0.307*	− 0.72 ± 0.39*
MOD (*n* = 11)	0.85 ± 0.16	0.764 ± 0.133	1.27 ± 0.24	0.12 ± 0.02	0.11 ± 0.02	0.796 ± 0.331	− 1.00 ± 0.49

Values are mean ± SD

*LOW* low intensity (45% *V*O_2max_); *MOD* moderate intensity (65% *V*O_2max_)

*p < 0.05, LOW vs. MOD

### Dietary intake

In the 48 h preceding the trials, there were no significant differences between LOW and MOD (paired t test analysis) in the intake of energy (3699 ± 770 kcal vs. 3949 ± 997 kcal; *p* = 0.27, ES = 0.28), carbohydrate (420 ± 136 g vs. 462 ± 174 g; *p* = 0.28, ES = 0.27), protein (185 ± 38 g vs. 185 ± 45 g; *p* = 0.94, ES = 0.02), fat (143 ± 37 g vs. 148 ± 41 g; *p* = 0.58, ES = 0.14), Na^+^ (5970 ± 2588 mg vs. 5965 ± 2481 mg; *p* = 0.99, ES = 0.002), K^+^ (4601 ± 1436 mg vs. 3965 ± 1573 mg; *p* = 0.16, ES = 0.42), or water (4.7 ± 1.9 L vs. 4.4 ± 1.7 L; *p* = 0.64, ES = 0.15).

### Absolute sweat electrolyte concentrations and sweating rate

**Fig. 2:**
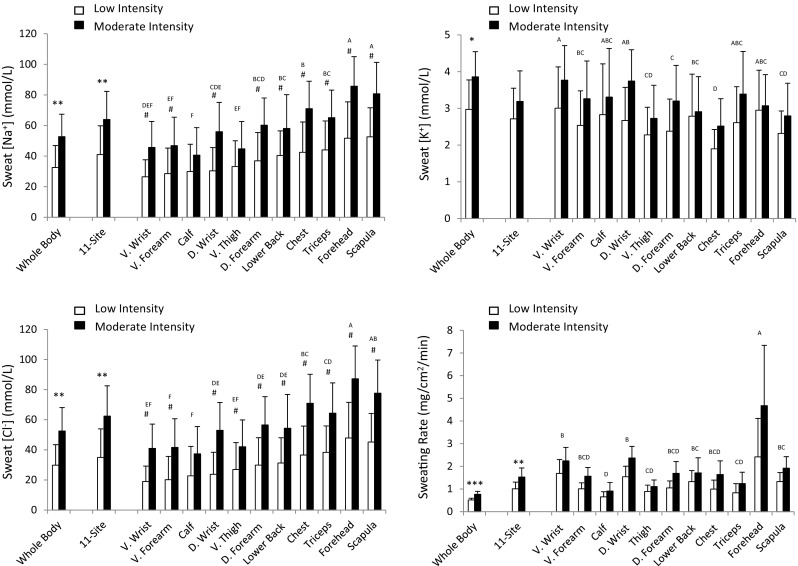
Sweat sodium, chloride, and potassium concentrations and sweating rate during LOW and MOD intensity trials. *n* = 11 for all sites except D. Wrist and V. Wrist, where *n* = 10. *D* dorsal, *V* ventral. *p < 0.05, **p < 0.01, ***p < 0.001, LOW vs. MOD for whole body and 11-site aggregate (paired *t* tests). There was a significant main effect of intensity, region, and interaction on sweat [Na^+^] and [Cl^−^]. For sweat [K^+^] and RSR, there was a significant main effect of intensity and region, but no interaction. ^#^*p* < 0.05, LOW vs. MOD within sites (after Tukey’s post hoc adjustment for multiple comparisons). Letters indicate regional differences collapsed across intensities (sites sharing same letter are not significantly different)

Figure 2 shows sweat [Na^+^], [Cl^−^], and [K^+^] and SR during LOW and MOD trials. WB and 11-site aggregate REG sweat electrolyte concentrations and SR were lower during LOW vs. MOD trials. There was a significant main effect of intensity (*p* < 0.0001), main effect of region (*p* < 0.0001), and interaction (*p* < 0.001) on sweat [Na^+^] and [Cl^−^]. Post hoc results showed that REG sweat [Na^+^] increased from LOW to MOD at all sites except the ventral thigh and calf. REG sweat [Cl^−^] increased from LOW to MOD at all sites except the calf. Log transformation was required for the sweat [K^+^] and RSR data prior to ANOVA. There was a significant main effect of intensity (*p* < 0.0001) and main effect of region (*p* < 0.0001), but no interaction on sweat [K^+^] (*p* = 0.20) and RSR (*p* = 0.52).

For sweat [Na^+^] and [Cl^−^], the ES for the difference between LOW and MOD were moderate for calf ([Na^+^]: 0.58; [Cl^−^]: 0.74) and thigh ([Na^+^]: 0.65; [Cl^−^]: 0.79), but large for all other sites ([Na^+^]: 0.84–1.24; [Cl^−^]: 1.02–1.34), including 11-site aggregate ([Na^+^]: 1.06; [Cl^−^]: 1.16). LOW vs. MOD intensity ES for sweat [K^+^] were generally smaller, with low ES for the forehead and lower back (0.12–0.13), moderate ES for ventral wrist, ventral forearm, triceps, scapula, calf, thigh, and 11-site (0.36–0.71), and high ES for dorsal wrist, dorsal forearm, and chest (0.83–1.06). LOW vs. MOD intensity ES for RSR was moderate for lower back (0.63), thigh (0.73), and calf (0.78), and high for all other sites (0.83–1.30), including 11-site aggregate (1.20). The ES for the difference between intensities was high for WB sweat [Na^+^] (1.15), [K^+^] (1.03), and [Cl^−^] (1.23) as well as WBSR (1.50).

### Total sweat electrolyte losses

**Fig. 3:**
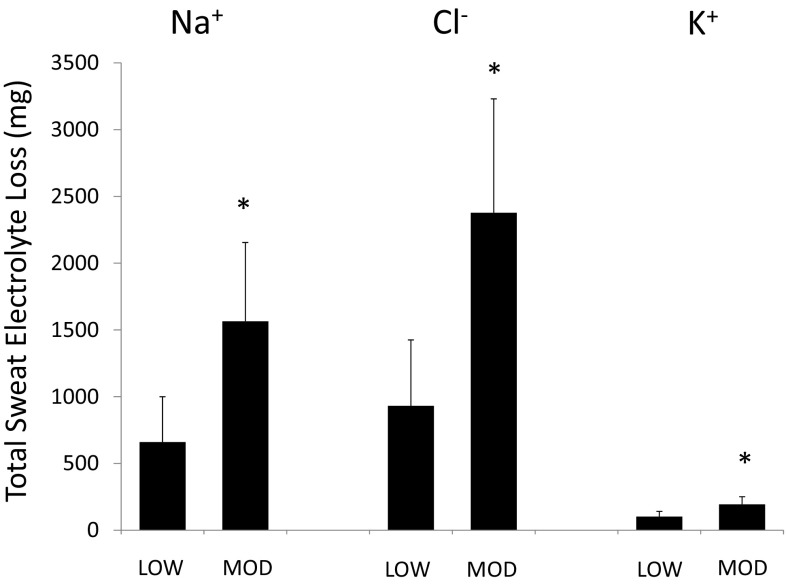
Total sweat sodium, chloride, and potassium losses during LOW and MOD intensity trials. *p < 0.05, LOW vs. MOD

Figure 3 shows total sweat electrolyte losses based on WB measurements during the 90 min of exercise. Total losses were significantly lower during LOW vs. MOD for sweat Na^+^ (659 ± 340 mg vs. 1565 ± 590 mg; *p* < 0.0001, ES = 1.37), sweat Cl^−^ (931 ± 494 mg vs. 2378 ± 853 mg; *p* < 0.0001, ES = 1.44), and sweat K^+^ (102 ± 39 mg vs. 194 ± 58 mg; *p* < 0.0001, ES = 1.37). Total sweat Na^+^ loss was lower than 24-h dietary Na^+^ intake for both LOW (2985 ± 1294 mg/day; *p* < 0.0001, ES = 1.54) and MOD (2982 ± 1240 mg/day; *p* = 0.002, ES = 1.19).

### Relation between regional and whole body

**Fig. 4:**
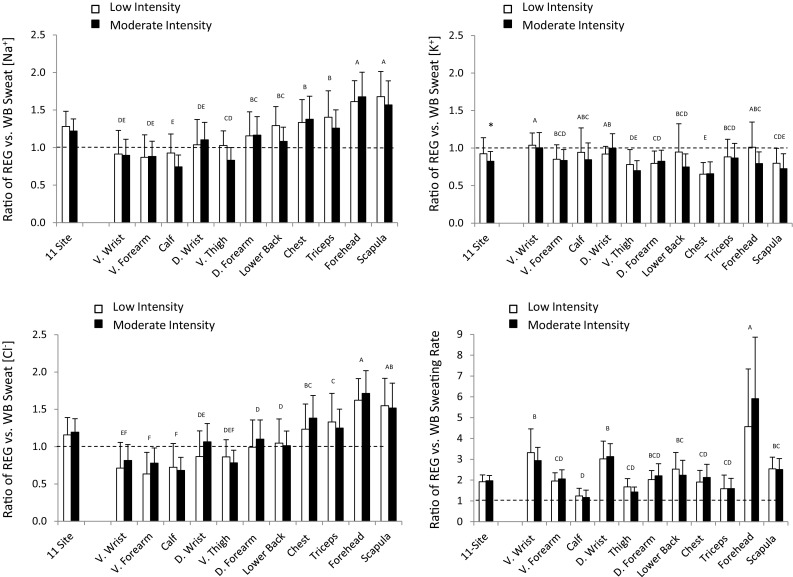
Ratio between regional and whole-body measures of sweat sodium, chloride, and potassium concentrations as well as sweating rate during LOW and MOD intensity trials. *n* = 11 for all sites except D. Wrist and V. Wrist, where *n* = 10. *D* dorsal, *V* ventral. *p < 0.05, LOW vs. MOD for 11-site aggregate (paired t tests). There was a significant main effect of intensity and region, but no interaction on the sweat [Na^+^], [Cl^−^], and [K^+^] ratios. There was a significant main effect of region, but no effect of intensity or interaction on the sweating rate ratio. Letters indicate regional differences collapsed across LOW and MOD (sites sharing same letter are not significantly different). Dashed line indicates ratio of 1.0 (regional = whole body)

Figure 4 shows results for the ratio between REG and WB measures of sweat [Na^+^], [K^+^], and [Cl^−^] as well as SR during LOW and MOD trials. The 11-site aggregate comparison between LOW and MOD trials was not significant for sweat [Na^+^] (*p* = 0.31), sweat [Cl^−^] (*p* = 0.58), or SR (*p* = 0.50), but was significant for sweat [K^+^] (*p* = 0.03). There was a significant main effect of intensity (*p* = 0.04) and main effect of region (*p* < 0.0001), but no intensity-by-region interaction (*p* = 0.24) on the sweat [Na^+^] ratio. Log transformation was required for the sweat [Cl^−^], [K^+^], and RSR ratio data prior to ANOVA. There was a significant main effect of region (*p* < 0.0001) and intensity (*p* = 0.01), but no intensity-by-region interaction (*p* = 0.47) on the sweat [Cl^−^] ratio. There was a main effect of intensity (*p* < 0.01) and main effect of region (*p* < 0.0001), but no intensity-by-region interaction (*p* = 0.06) on the sweat [K^+^] ratio. There was a main effect of region (*p* < 0.0001), but no effect of intensity (*p* = 0.53) or intensity-by-region interaction (*p* = 0.36) on the SR ratio.

The ES for the difference between LOW and MOD for REG-to-WB sweat [Na^+^] ratio was low for ventral wrist, dorsal forearm, ventral forearm, and chest (0.03–0.15); moderate for dorsal wrist, triceps, scapula, forehead, and 11-site (0.21–0.48); high for lower back, thigh, and calf (0.81–0.96). ES for the REG-to-WB ratio for sweat [Cl^−^] was in the low (0.09–0.18; scapula, lower back, calf, and 11-site)-to-moderate (0.25–0.65; all other sites) range. Likewise, ES for the REG-to-WB ratio for sweat [K^+^] was in the low (0.04–0.19; ventral wrist, dorsal forearm, ventral forearm, triceps, and chest)-to-moderate (0.35–0.78; all other sites) range. Finally, LOW vs. MOD intensity ES for the RSR-to-WBSR ratio were low for dorsal wrist, triceps, scapula, and 11-site (0.01–0.17), and moderate for all other sites (0.22–0.72).

Table 2 shows the linear regression results and final prediction models for REG vs. WB sweat [Na^+^], [Cl^−^], and sweating rate. Based on these results, Table 2 lists the final suggested models for the prediction of WB from REG. Where there were significant effects of intensity or intensity–REG interactions, separate models are needed for LOW and MOD. Where intensity had no effect on the prediction of WB from REG, the data can be collapsed into one (reduced) model and used irrespective of intensity. Table 2 also shows how the slope and y-intercept of the regression lines compared against the line of identity. The regression lines were not different than the line of identity when predicting WB sweat [Na^+^] and [Cl^−^] from the dorsal forearm. However, for most other REG sites, slope and intercept were significantly different than one and zero, respectively.

**Table 2:** Linear regression results and final prediction models for regional vs. whole-body sweat [Na^+^], sweat [Cl^−^], and sweating rate

	Final model(s), where *y* = whole body, *x* = regional (*r*^2^ value for final model)					
	Sweat [Na^+^]		Sweat [Cl^−^]		Sweating rate	
Dorsal wrist		*y* = 0.77*x* + 16.5*^#^ (*r*^2^ = 0.95)		*y* = 0.76*x* + 18.2*^#^ (*r*^2^ = 0.94)		*y* = 0.274*x* + 0.157* (*r*^2^ = 0.91)
Ventral wrist	^†^	LOW: *y* = 0.70*x* + 11.1 (*r*^2^ = 0.49) MOD: *y* = 0.66*x* + 21.3 (*r*^2^ = 0.58)	^†^	LOW: *y* = 0.66*x* + 14.4^#^ (*r*^2^ = 0.44) MOD: *y* = 0.67*x* + 23.0*^#^ (*r*^2^ = 0.54)	^‡^	LOW: *y* = 0.032*x* + 0.461*^#^ (*r*^2^ = 0.06) MOD: *y* = 0.152*x* + 0.427*^#^ (*r*^2^ = 0.42)
Dorsal forearm		*y* = 0.86*x* + 8.1 (*r*^2^ = 0.97)		*y* = 0.85*x* + 11.6 (*r*^2^ = 0.97)		*y* = 0.353*x* + 0.199*^#^ (*r*^2^ = 0.90)
Ventral forearm	^‡^	LOW: *y* = 0.74*x* + 11.3^#^ (*r*^2^ = 0.75) MOD: *y* = 0.64*x* + 22.9*^#^ (*r*^2^ = 0.65)		*y* = 1.02*x* + 12.1^#^ (*r*^2^ = 0.97)		*y* = 0.400*x* + 0.218*^#^ (*r*^2^ = 0.91)
Triceps	^†^	LOW: *y* = 0.65*x* + 4.0* (*r*^2^ = 0.74) MOD: *y* = 0.56*x* + 16.4 (*r*^2^ = 0.47)		*y* = 0.79*x* + 16.6*^#^ (*r*^2^ = 0.95)	^†^	LOW: *y* = 0.105*x* + 0.429*^#^ (*r*^2^ = 0.30) MOD: *y* = 0.204*x* + 0.512*^#^ (*r*^2^ = 0.59)
Chest	^†^	LOW: *y* = 0.61*x* + 6.5* (*r*^2^ = 0.72) MOD: *y* = 0.59*x* + 11.2 (*r*^2^ = 0.52)		*y* = 0.63*x* + 22.9*^#^ (*r*^2^ = 0.97)	^†^	LOW: *y* = 0.119*x* + 0.397*^#^ (*r*^2^ = 0.38) MOD: *y* = 0.123*x* + 0.563*^#^ (*r*^2^ = 0.31)
Scapula		*y* = 0.67*x* + 9.4* (*r*^2^ = 0.96)		*y* = 0.66*x* + 10.8* (*r*^2^ = 0.96)	^†^	LOW: *y* = 0.145*x* + 0.324*^#^ (*r*^2^ = 0.55) MOD: *y* = 0.162*x* + 0.454*^#^ (*r*^2^ = 0.38)
Lower back	^‡^	LOW: *y* = 0.78*x* + 0.9 (*r*^2^ = 0.78) MOD: *y* = 0.60*x* + 18.0*^#^ (*r*^2^ = 0.82)	^†^	LOW: *y* = 0.72*x* + 7.2 (*r*^2^ = 0.79) MOD: *y* = 0.62*x* + 19.0*^#^ (*r*^2^ = 0.79)	^†^	LOW: *y* = 0.091*x* + 0.395*^#^ (*r*^2^ = 0.33) MOD: *y* = 0.090*x* + 0.611*^#^ (*r*^2^ = 0.20)
Ventral thigh	^†^	LOW: *y* = 0.77*x* + 7.0 (*r*^2^ = 0.82) MOD: *y* = 0.73*x* + 20.3^#^ (*r*^2^ = 0.78)	^†^	LOW: *y* = 0.70*x* + 11.0^#^ (*r*^2^ = 0.84) MOD: *y* = 0.76*x* + 20.5*^#^ (*r*^2^ = 0.76)	^†^	LOW: *y* = 0.244*x* + 0.299*^#^ (*r*^2^ = 0.81) MOD: *y* = 0.405*x* + 0.316*^#^ (*r*^2^ = 0.78)
Calf	^†^	LOW: *y* = 0.68*x* + 12.3^#^ (*r*^2^ = 0.71) MOD: *y* = 0.75*x* + 22.1*^#^ (*r*^2^ = 0.86)	^†^	LOW: *y* = 0.57*x* + 16.8*^#^ (*r*^2^ = 0.67) MOD: *y* = 0.77*x* + 23.8*^#^ (*r*^2^ = 0.84)	^†^	LOW: *y* = 0.245*x* + 0.356*^#^ (*r*^2^ = 0.52) MOD: *y* = 0.267*x* + 0.522*^#^ (*r*^2^ = 0.56)
Forehead		*y* = 0.55*x* + 22.2*^#^ (*r*^2^ = 0.95)		*y* = 0.56*x* + 18.8*^#^ (*r*^2^ = 0.97)	^†^	LOW: *y* = 0.022*x* + 0.463*^#^ (*r*^2^ = 0.24) MOD: *y* = 0.031*x* + 0.620*^#^ (*r*^2^ = 0.38)
11-Site		*y* = 0.84*x* + 8.3* (*r*^2^ = 0.98)		*y* = 0.80*x* + 10.8*^#^ (*r*^2^ = 0.98)		*y* = 0.460*x* + 0.001* (*r*^2^ = 0.96)

^†^*p* < 0.05, intensity

^‡^*p* < 0.05, intensity–REG interaction

*Slope ≠ 1; # y-intercept ≠ 0. LOW, low intensity (45% *V*O_2max_); MOD, moderate intensity (65% *V*O_2max_)

There was a significant correlation between REG and WB at all the sites for sweat [Na^+^] (LOW: *r* = 0.70–0.92, *p* < 0.05; MOD: *r* = 0.68–0.93, *p* < 0.05) and [Cl^−^] (LOW: *r* = 0.66–0.93, *p* < 0.05; MOD: *r* = 0.68–0.89, *p* < 0.05). For sweat [K^+^] there was a significant REG-WB correlation at most sites (LOW: *r* = 0.64–0.94, *p* < 0.05; MOD: *r* = 0.64–0.82, *p* < 0.05) with the exception of forehead, lower back, and thigh at LOW (*r* = 0.55–0.56, *p* = 0.07–0.08) and chest and scapula at MOD (*r* = 0.46, *p* = 0.15–0.16). With respect to SR, at LOW intensity, the REG-WB correlation at the dorsal and ventral wrist, lower back, triceps, and ventral forearm sites was not significant (*r* = 0.24–0.57, *p* = 0.06–0.51), but was significant at the remaining sites (*r* = 0.62–0.90, *p* < 0.05). During MOD, there was a significant REG-WB correlation for SR at most sites (*r* = 0.61–0.95, *p* < 0.05) with the exception of chest and lower back (*r* = 0.45–0.56, *p* = 0.08–0.16).

### Sites most suitable for predicting whole-body sweating responses

Table 3 shows a heat map with a summary of results to help determine the most suitable sites, if any, for using REG measures to predict WB sweat [Na^+^], [Cl^−^], and SR. For the purpose of this study, criteria were set as: (1) significant and meaningful (i.e., large ES) increase in absolute REG values from LOW to MOD; (2) no significant or meaningful difference in the REG-to-WB ratio value between LOW and MOD; (3) no effect of intensity or intensity–REG interaction effect on the regression model predicting WB from REG. The dorsal forearm, dorsal wrist, scapula, forehead, and 11-site aggregate met all criteria; whereas the lower back, ventral thigh, and calf met the fewest criteria for sweat [Na^+^] and [Cl^−^]. The results also indicate that there are more sites meeting the criteria for [Cl^−^] (7 individual sites and 11-site aggregate) than sweat [Na^+^] (4 individual sites and 11-site aggregate). For sweat [K^+^], the dorsal wrist was the only site that met all criteria; while the dorsal forearm and chest met most criteria, and all other sites only met one criterion.

**Table 3:**
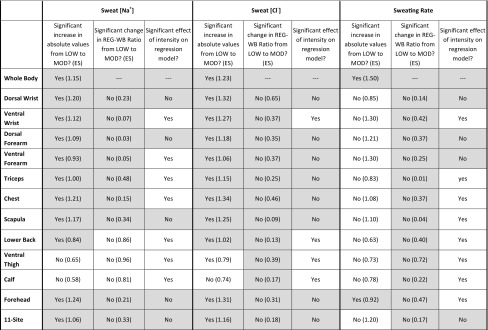
Heat map to determine most suitable sites for using regional measures to predict whole-body sweat [Na^+^], sweat [Cl^−^], and sweating rate

Gray areas indicate where the following criteria were met: (1) mean difference in absolute REG value between LOW and MOD *p* < 0.05 (ANOVA followed by post hoc analysis) and large effect size (definitions per Cohen 1988: small: <0.2, moderate: 0.2–0.8, large: > 0.8); (2) mean difference in REG-to-WB ratio value between LOW and MOD *p* > 0.05 and small-moderate effect size; (3) intensity or intensity–regional interaction effect *p* > 0.05 on the regression model predicting WB from REG

*ES* effect size, *REG* regional, *WB* whole body, *LOW* low intensity (45% *V*O_2max_), *MOD* moderate intensity (65% *V*O_2max_)

## Discussion

In this study, REG and WB sweat electrolyte concentrations were measured during LOW and MOD relative exercise intensities to determine the effect of increasing workload on WB sweat electrolyte concentrations, total sweat electrolyte losses, and the relation between REG and WB sweat electrolyte concentrations. It was found that WB sweat [Na^+^], [Cl^−^], and [K^+^] increased by 62%, 76%, and 30%, respectively, from LOW to MOD intensity exercise that elicited a 48% increase in WBSR. This equated to an increase in total sweat Na^+^ and Cl^−^ loss of 137% and 155%, respectively, with just a 60 W (277 kcal) difference in cycling workload; which may impact significantly on fluid/electrolyte intake strategies for athletes between workouts (e.g., going from training to competition). Another significant finding was that, while most REG sites exhibited a significant increase in electrolyte concentrations from LOW to MOD, some sites (thigh and calf) did not. Furthermore, the impact of intensity on the regression model predicting WB sweat electrolyte concentrations from REG was site-dependent. These results have practical application in determining sweat testing best practices. That is, regression equations may be useful in predicting WB sweat [Na^+^] and [Cl^−^] from some REG sites (e.g., dorsal forearm) irrespective of intensity when exercising between 45 and 65% *V*O_2max_, but other sites (especially ventral wrist, lower back, thigh, and calf) require separate prediction equations that account for workload.

The novel finding of increased WB sweat electrolyte concentrations in the present study, although not surprising, is practically significant, since the WBW method provides a more direct measurement to calculate athletes’ total sweat electrolyte losses (when coupled with WBSR) and better informs intake strategies than REG measurements. It has been recommended by several independent investigators and national organizations that assessment of sweat electrolyte losses should be conducted to help determine the need for and nature of individualized electrolyte replacement plans (Lewis et al. 2018; Maughan and Shirreffs 2008; McDermott et al. 2017; Sawka et al. 2007; Shirreffs and Sawka 2011; Thomas et al. 2016). Sodium intake during training/competition is recommended in athletes with high sweating rates and “salty sweat”, especially when exercise exceeds 2 h (Coyle 2004; McDermott et al. 2017; Shirreffs and Sawka 2011; Thomas et al. 2016). Based on the mean data, if the “average” athlete from the present study exercises for 2 h, their total sweat Na^+^ loss would be ~ 0.9 g at LOW versus ~ 2.1 g at MOD. This simple example illustrates how changes in exercise intensity potentially alter the importance or necessity of electrolyte replacement for a given individual. And given the relatively low WBSRs in the present study (< 1 L/h), it is important to note that significantly higher sweat Na^+^ losses (e.g., over 3–4 g) (Coyle 2004; Shirreffs and Sawka 2011) are plausible, especially in athletes who are larger, wearing protective gear, or exercising in warmer conditions than that of the present study.

Previous studies have reported increases in REG sweat [Na^+^] with a change in exercise or environmental conditions that elicit an increase in sweating rate (Buono et al. 2008; Dziedzic et al. 2014; Yoshida et al. 2006). Buono et al. (2008) found that ventral forearm sweat [Na^+^] ranged from 19 to 59 mmol/L during incremental exercise from 50 to 90% HR_max_. In Buono et al.’s intensity range most similar to the present study (60–80% HR_max_), sweat [Na^+^] increased from ~ 30 to ~ 47 mmol/L (Buono et al. 2008). Similarly, ventral forearm sweat [Na^+^] increased from 28.5 to 46.8 mmol/L in the present study. In addition, Yoshida et al. (2006) found an increase in chest sweat [Na^+^] when increasing intensity from 40% to 60% *V*O_2max_ and Dziedzic et al. (2014) reported a significant increase in dorsal forearm sweat [Na^+^] (60 to 69 mmol/L) between temperate (21 °C) and hot (32 °C) conditions. Thus, our findings corroborate the results of previous studies reporting increased forearm and chest sweat [Na^+^] with increased intensity/heat stress and extend these findings to several additional upper body sites (wrists, triceps, scapula, lower back, and forehead).

Another novel finding of the present study was the lack of a significant increase in sweat [Na^+^] on the lower body (thigh and calf). While there were non-significant increases at these sites, the percentage increase (35–36%) was lower than all other sites studied (44–85%). Sweat [Cl^−^] results followed a similar pattern to that of [Na^+^]. In line with the electrolyte results, RSR also increased to a lesser extent on the lower body (25–39%) than most sites on the upper body (44–94%), with the exception of ventral wrist and lower back (29–33%). This finding is consistent with the previous studies; such as that of Smith and Havenith (2011), who found a 32–39% increase in RSR on the ventral thigh and calf compared with 50–130% on upper body sites (in common with present study), with the exception of the lower back (30%); when increasing intensity from 55 to 75% *V*O_2max_.

The physiological explanation for the upper vs. lower body differences in response to increased intensity in the present study is difficult to define, since body core temperature and local skin temperature and blood flow were not measured. Perhaps, some insights may be gleaned from Smith and Havenith (2011), who found that, while an increase in intensity from 55 to 75% *V*O_2max_ was associated with an elevation in body core temperature by 0.38 °C, sudomotor sensitivity decreased on the thigh, calf, and lower back (by 14–49%). By contrast, at most upper body sites, sudomotor sensitivity increased (by 23–171% at sites in common with the present study) with increased workload (Smith and Havenith 2011). Incidentally, REG differences in SR were not related to local skin temperature (Smith and Havenith 2011). Mechanism notwithstanding, the thigh and calf may not be the best sites for detecting differences in sweat electrolyte concentrations across various exercise intensities; at least not for recreationally trained, non-heat acclimated subjects (as tested in the present study). It is thought that training and heat acclimation may affect the REG distribution of SR, and by extension sweat electrolyte concentrations. However, results to date have been equivocal (Hofler 1968; Patterson et al. 2004; Regan et al. 1996; Shvartz et al. 1979), and therefore, the effect of training and heat acclimation status on REG sweat distribution and REG-to-WB relations, especially with regards to electrolyte concentrations, requires further research.

Another factor that can impact sweat [Na^+^] is dietary Na^+^ intake (Armstrong et al. 1985; Hargreaves et al. 1989); thus, subjects in the present study were asked to eat a consistent diet 48 h prior to each trial (Na^+^ intake was 2985 mg/day and 2983 mg/day for the LOW and MOD trials, respectively) to minimize the potential effect of acute dietary changes on study results. Nonetheless, more research is needed to understand how dietary Na^+^ intake impacts REG and WB sweat [Na^+^]. While some studies have found significant increases in sweat [Na^+^] with controlled high Na^+^ diets (Armstrong et al. 1985; Hargreaves et al. 1989), others have found no differences (Koenders et al. 2017; Konikoff et al. 1986). As discussed in a recent systematic review (McCubbin and Costa 2018), the discrepancy between studies may be due in part to methodological differences in sweat collection. Furthermore, as most previous studies measured sweat at either the REG or WB level (not both), the effect of dietary Na^+^ intake on the relation between REG and WB is uncertain.

In agreement with previous studies, we found that, while there was a significant correlation between REG and WB sweat [Na^+^] and [Cl^−^], the line of regression was significantly different than the line of identity for most sites. This suggests, as expected, that REG measurements cannot be used as a direct surrogate for WB sweat electrolyte concentrations. Rather, regression equations can be used to provide a prediction of WB sweat [Na^+^] and [Cl^−^]. The reason for the overestimation of SR and electrolyte concentrations from most REG sites relative to that of WB is probably due to a number of factors (Baker 2017), including the anatomical location of the REG sites used in the present study. Patch locations were chosen based on practicality (i.e., sites that are accessible and more likely to be used in real-world settings) and consistent with previous research, especially field-based studies. Therefore, other anatomical sites, which are known to have relatively low sweating rates and sweat electrolyte concentrations, such as the feet, hands (palmar surface), parts of the face (cheeks), and scalp, were not used in this study (Patterson et al. 2000; Smith and Havenith 2011; Taylor and Machado-Moreira 2013). Although speculative, if these sites were included in the REG measures, it is possible that the surface area-weighted aggregate REG SR and sweat electrolyte concentrations would be more similar to that of WB.

The novel aspect of this study was to determine if the regression equations used to predict WB from REG differ from LOW to MOD intensity. We found that the impact of intensity on the relation between REG and WB was dependent upon the site. For instance, at most sites, sweat electrolyte concentration increased proportionally, such that the REG-to-WB ratio did not differ between LOW and MOD (Fig. 4). However, for some sites (lower back, thigh, and calf), the REG-to-WB ratio for sweat [Na^+^] was lower during MOD than LOW intensity (Table 3), and in turn, the regression model predicting WB from REG was significantly impacted by intensity (Table 2). Table 3 provides a summary of all results to help determine which sites are more suitable for predicting WB sweat [Na^+^] and [Cl^−^]. Based on this heat map, the dorsal wrist, dorsal forearm, scapula, forehead, and 11-site aggregate are most suitable, while the lower back, thigh, and calf are least suitable for predicting WB sweat [Na^+^] and [Cl^−^] from REG at multiple workloads. The heat map results suggest that additional sites may be suitable for predicting WB sweat [Cl^−^] (ventral forearm, triceps, and chest). The suggested final models are shown in Table 2; however, it is important to note that these regression equations are only applicable when similar methods are used, since variations in sweat collection and sample analysis may impact results.

Overall, the results of this study demonstrate the importance of carefully considering the methodology (REG site) and the conditions (exercise intensity) in which the sweat tests are conducted. For example, the sweating response differs significantly between exercise-heat stress and pharmacologically induced sweating (e.g., pilocarpine iontophoresis) (di Sant’Agnese and Powell 1962; Hjortskov et al. 1995; Sato et al. 1970; Schwachman and Antonowicz 1962; Vimieiro-Gomes et al. 2005). In addition, estimating total sweat electrolyte losses from sweating rate alone (Taylor and Machado-Moreira 2013) is unsubstantiated; as data from this and previous studies (Baker et al. 2018) suggest that only ~ 35–50% of the interindividual variation in total sweat Na^+^ or Cl^−^ loss is explained by WBSR. When knowledge of an athlete’s sweat electrolyte concentration or total electrolyte loss is desired, it is best practice to make direct measurements, conducting individualized tests during exercise and in conditions specific to their sport (Baker 2017).

Sweat [K^+^] was included in this study, because it has been suggested to be a good measure of quality control (Baker 2017; Dziedzic et al. 2014; Weschler 2008), since it is expected to remain relatively constant and similar to that of blood (~ 3–5 mmol/L) despite changes in RSR. We found that sweat [K^+^] increased significantly for WB (from 3.0–3.9 mmol/L) and 11-site REG aggregate (2.7 to 3.2 mmol/L) from LOW to MOD. Although this increase in sweat [K^+^] was fairly consistent across subjects, it was a relatively small increase and remained well within normal range. Thus, our findings suggest that exercise intensity has only a trivial impact on sweat K^+^ losses in practice.

## Limitations and future directions

RSR vs. WBSR data are shown in all the tables and figures for comparison with electrolyte results. There are limitations with the absorbent patch technique: as the microenvironment (increased skin temperature) created by the occlusive dressing can alter (increase) REG sweat flow rate (albeit skin temperature was not measured in this study). Thus, prediction of WBSR from RSR or other methods is tenuous to date, and the best practice remains more direct methods based on body mass change and measurements of fluid/mass input and output.

In this study, workload was based on relative exercise intensity for purposes of practical applicability. However, future studies, particularly those interested in investigating mechanisms underlying differences in WB sweat electrolyte concentrations, should consider standardizing absolute workload and include other measurements such as body core temperature, skin temperature, skin blood flow, and/or heat-activated sweat gland density.

The results reported here are only applicable to the specific conditions of the present study. It is possible that various factors not accounted for, in this study, could impact the relation between REG and WB sweat electrolyte concentrations. Therefore, future research is needed to determine the effect of patch application timing and adherence duration, exercise mode (e.g., involving running or intermittent sports), variations in environmental conditions (e.g., ambient temperature, humidity, wind, and solar load), and individual factors (e.g., race/ethnicity, training, and heat acclimation status) on the relation between REG and WB sweat electrolyte concentrations.

## Conclusion

Total sweat Na^+^ and Cl^−^ losses increased by ~ 150% from 45 to 65% *V*O_2max_, suggesting that different fluid/electrolyte intake strategies may be needed depending upon variations in exercise intensity between training sessions/competitions. The effect of exercise intensity on the relation between REG and WB sweat electrolyte concentrations varied among sites. Based on these findings, it seems that regression equations can be used to predict WB sweat [Na^+^] and [Cl^−^] from some REG sites (e.g., dorsal forearm) irrespective of intensity when exercising between 45% and 65% *V*O_2max_ and using the same methodology employed in this study. However, other sites (especially the ventral wrist, lower back, thigh, and calf) require separate prediction equations that account for workload.

## Abbreviations

Cl^−^: Chloride
CV: Coefficient of variation
ECG: Electrocardiogram
ES: Effect size
HR_max_: Maximal heart rate
K^+^: Potassium
LOW: Low intensity
MOD: Moderate intensity
Na^+^: Sodium
REG: Regional
RPE: Rating of perceived exertion
RSR: Regional sweating rate
USG: Urine specific gravity
*V*O_2max_: Maximal oxygen uptake
WB: Whole body
WBSL: Whole-body sweat loss
WBSR: Whole-body sweating rate
WBW: Whole-body washdown
